# Multiwave Locked System Laser Treatment Reduces the Bacterial Load in the Gingival Sulcus of Dogs After Plaque Removal

**DOI:** 10.3390/vetsci12080767

**Published:** 2025-08-16

**Authors:** Ivana Pallante, Paolo Squarzoni, Elisa Mazzotta, Nicola Pozzato, Monica Monici

**Affiliations:** 1 Veterinary Forensic Medicine Unit, Istituto Zooprofilattico Sperimentale delle Venezie (IZSVE), 36100 Vicenza, Italy; ivanapallante95@live.it (I.P.); emazzotta@izsvenezie.it (E.M.); 2 Independent Veterinary Practitioner, 40062 Molinella, Italy; vetsquarzoni@gmail.com; 3 ASA Campus Joint Laboratory, ASA Research Division, Department of Experimental and Clinical Biomedical Sciences, University of Florence, 50134 Florence, Italy; monica.monici@unifi.it

**Keywords:** dog, periodontal disease, prevention, bacterial load, NIR laser, laser therapy

## Abstract

Periodontal disease is a prevalent condition in companion animals. It is crucial to prevent the plaque and bacteria on tooth surfaces to avoid gingivitis and the more harmful periodontitis. The aim of the present study was to assess the impact of MLS laser treatment on the bacterial load by analyzing the gingival swabs of a total of 16 owned dogs with no history of dental disease that were selected from a cohort of patients admitted for plaque removal and dental hygiene procedures. Following each dental hygiene session, each dog received a single MLS laser therapy treatment (808–905 nm diode laser, frequency 36 Hz, and fluence 0.16 J/cm^2^). Swabs were collected from the two upper premolars before and after the laser treatment. These were submitted for mesophilic bacteria counts, and microbiological analysis was conducted on 10 positive cultures to evaluate the changes in the oral bacterial microbiota. MLS laser therapy statistically significantly reduced the mesophilic bacteria count by 1 log, with higher pre-laser treatment counts (n = 0.44; 5.77 ± 0.21 95%CI) in comparison to post-laser treatment counts (n = 0.73; 4.80 ± 0.346 95%CI). The MLS laser therapy was found to reduce the bacterial load in 80% of the subjects (*p* < 0.05). No significant differences pre- and post-laser treatment were observed in the bacteria species isolated from the microbiological cultures. MLS laser therapy appears to be a useful, non-invasive method for reducing the bacterial load in the treatment and prophylaxis of periodontal disease in dogs.

## Simple Summary

The prevention and control of periodontal diseases in dogs can become challenging in the absence of veterinary intervention if the requested home-based daily care is not guaranteed. To evaluate the eventual efficacy of Multiwave Locked System (MLS) laser application in conjunction with the standard plaque removal procedure, in this study, the comparative analysis of mesophilic oral bacteria via gingival swabs in sixteen dogs was executed. The laser application resulted in a 1-log reduction in bacteria counts and no significant differences in bacteria species isolated from gingival swabs. The MLS laser application was found to be effective in 80% of subjects (*p* < 0.05), demonstrating its potential as a valuable tool in the prevention of periodontal disease, particularly given its simplicity and non-invasive nature.

## 1. Introduction

Periodontal disease encompasses several pathological conditions, including gingivitis and periodontitis [1]. Primary care veterinary practices, where diagnosis of periodontal disease is mainly based on visual oral assessment of conscious dogs, report an average prevalence of 9.3 to 18.2% within the dog population, whereas the prevalence increases to between 44 and 100% after detailed examinations of anesthetized dogs [2]. In addition, in the early stages, periodontal diseases occur without overt clinical symptoms, such as pain or discomfort, and thus they are frequently diagnosed at an advanced stage [3,4]. In companion animals, there is evidence that predisposition is linked to breed and size. Therefore, small and toy breeds of dogs (e.g., Yorkshire Terriers, Maltese, etc.) [5,6,7] and brachycephalic breeds (e.g., Pugs) appear to be more susceptible to the disease [8]. Diet, behavior, environment, and genetics are also likely to play a role in the development of periodontal disease [2]. The primary factor contributing to periodontal disease is inadequate oral hygiene, which allows bacterial plaque to accumulate on the tooth surface [9,10,11,12]. In healthy subjects, the accumulation of bacterial plaque on the surface of teeth, in the gingival sulcus, and on the gingiva is controlled by physiological defense mechanisms. These include the integrity of the epithelium, the washing action of saliva, tongue and lip movements, chewing, and the action of bacteriostatic and bactericidal substances in the gingival fluid [13].

When these mechanisms do not work or work only partially, periodontal disease arises. Periodontal disease is a chronic, recurrent, and continuously progressing pathology. In the acute stages, the inflammatory response is characterized by the predominance of neutrophils and the subsequent formation of purulent exudate (dental abscess). If the pus finds a drainage route, the disease tends to enter the chronic phase, which is characterized by the presence of lymphocytes and plasma cells. The alternation of acute and chronic phases leads to the progressive destruction of the periodontal structures. The tissue response to the pathological presence of bacteria, which accumulate on the teeth and in the gingival sulcus, is an inflammatory reaction of the gum and gingival sulcus (gingivitis) or of all the structures that support the tooth (periodontitis).

It has been demonstrated that the tissue damage in periodontal diseases is a consequence of a dysregulated immune response to bacterial infection rather than being caused by the bacteria themselves [14]. Several studies have investigated bacterial species causing periodontal disease, and differing results were obtained by different authors. Davis et al. demonstrated that Gram-positive species predominated in dogs with periodontitis, while Gram-negative species were more abundant in subgingival samples collected from healthy dogs [15]. In contrast, other authors have reported that the transition from health to disease is characterized by a switch from the presence of mainly Gram-positive species to mainly anaerobic Gram-negative species [16].

The precise organisms involved in the initiation and evolution of the disease remain unknown [17]. One of the most abundant bacteria in the oral canine microbiome and with an important role in periodontal disease development is *Porphyromonas* spp., followed by *Peptostreptococcus canis*, an anaerobic Gram-positive species that is strongly linked to high-grade periodontitis in dogs [15,18,19,20]. Despite the continued challenge of preventing and controlling periodontal diseases, the most effective approach remains the management of the patient by the owner. Home care is the most important and effective method to prevent oral diseases, as it allows for the mechanical removal of bacterial plaque through daily dental brushing. This prevents the mineralization and subsequent transformation of plaque into tartar, which is not effectively removed through brushing [21]. However, it is not uncommon for home care to be poorly performed, inconsistent, or even ignored by the owner. Consequently, oral hygiene sessions under general anesthesia may be necessary for the ablation of plaque and supragingival tartar [22,23]. Over the past years, there has been a growing interest in additional treatments for the reduction in the bacterial load and improvement of gingival healing. In recent years, several studies have examined the efficacy of laser therapy in the treatment of periodontal disease in dogs [24,25]. Saglam et al. (2014) reported that a laser diode produces bactericidal and anti-inflammatory effects when it interacts with a periodontal pocket [26]. In veterinary dentistry, a recent study showed considerable elimination of bacteria after laser energy application in rats with induced periodontal disease [27].

In addition to the bacteria reduction, several studies reported that the important role of laser irradiation is the induction of photobiomodulation, which is used to accelerate the healing of various tissues and increase the number of cells and their vitality [28,29]. The laser application provokes a biostimulatory effect on bone tissue, contributing to an increase in the proliferation and differentiation of osteoblasts, which are the main cells for bone formation [30].

Recently, Multiwave Locked System (MLS) laser therapy, which differs from traditional lasers primarily in its use of synchronized, dual-wavelength emissions, has demonstrated growing research interest as a complementary, non-invasive therapeutic option for managing musculoskeletal disorders, chronic pain, wound healing, and neuropathic conditions [31]. This therapy is an alternative to standard lasers, which typically utilize a single wavelength source.

The aim of the present study was to assess the impact of MLS laser treatment on the bacterial load by analyzing the gingival swabs of owned dogs admitted to the clinic for standard plaque removal. A further explorative objective was to assess the bacteria species present in the swab before and after laser application.

## 2. Materials and Methods

Sixteen dogs without history of dental pathology and enrolled in this study were selected from patients with a standard oral hygiene appointment at Dr. Squarzoni Veterinary Clinic in Molinella (BO, Italy) between May 2019 and November 2020. Dogs that had undergone medical procedures and received antibiotics, immunomodulatory drugs, immunosuppressant, topical treatments, or laser therapy in the previous three months were excluded from the study. Prior to each dog’s enrollment, informed consent was obtained from all dog owners, for dogs who were deemed eligible to participate. Local ethics committee approval was not required, as the clinical study was conducted on biological samples collected non-invasively for diagnostic purposes during routine medical procedures that included the laser application. According to Yasuda L., the severity of periodontal conditions was evaluated as follows: (1) no significant findings; (2) mild periodontal disease—gingival swelling, gingival regression, and halitosis; (3) moderate periodontal disease—exposure of root, spontaneous bleeding, and tooth loss; and (4) severe periodontal disease—furcation involvement and fistula formation [32]. Following the professional dental scaling, a gingival swab was taken from the gingival sulcus of two upper 4th premolars for each dog before and after the laser treatment. The premolar teeth were selected because they have been reported to be the teeth most frequently affected by plaque deposition [8,33,34,35,36,37].

### 2.1. Laser Therapy

The laser treatments were conducted using a class IV NIR MLS laser with two synchronized sources (laser diodes) of model Mphi VET (ASA S.r.l., Vicenza, Italy). The two differ in wavelengths, peak power, and emission mode. The first laser diode is a pulsed device emitting at 905 nm with peak optical power of 25 W. Each pulse is composed of a pulse train (100 ns single pulse width; 90 kHz maximum frequency). The frequency of the pulse trains can be set in the range 1–2000 Hz, thus allowing the average power delivered to the tissue to be varied. The second laser diode (808 nm) can operate in continuous (power 1.1 W) or pulsed (repetition rate 1–2000 Hz) emission mode, with a 550 mW mean optical power output and a duty ratio of 50% independent of the repetition rate. The two laser beams operate in unison, in a synchronized manner, with their propagation axes coinciding. The laser treatment was administered using a headpiece held at a distance of a few millimeters from the tissue, with a spot of 2 cm diameter. Each treatment session comprised a single treatment point per tooth.

The instrument settings for the oral care treatment of dogs were as follows: wavelengths of 808 nm and 905 nm; duty cycle of 50%; frequency of 36 Hz; exposure time/treated point of 4 s; energy/point of 0.5 Joules; fluence of 0.16 J/cm^2^. These settings were in accordance with the manufacturer’s instructions and protocols.

### 2.2. Mesophilic Bacteria Count and Microbiological Culture

Swab samples for bacteria counts were collected by rubbing swabs on the gingival surface and then promptly placing them in Amies CLR medium; the tubes were stored at 4 °C until analysis. The swabs were processed for mesophilic bacteria count according to ISO 4833-1 and submitted for microbiological analyses that were performed on a subgroup of 10 dogs [38]. For the mesophilic bacteria count, the gingival swab was diluted in 3 mL of brain heart infusion (BHI) to create the stock solution. One milliliter of stock solution was transferred into 9 mL of tryptone solution (TS) to create the initial dilution which was then used to prepare the subsequent five dilutions. For each dilution, 1 mL was inoculated into 12–15 mL of liquefied plate count agar, and plates were then incubated at 30 °C ± 1 °C for 72 ± 3 h. After incubation, the number of bacterial colonies were counted in plates with a range of 10–300 colonies. Bacteria were cultured from each swab by inoculating 10 µL of stock solution (the swab homogenized in BHI, as described above) into two plates of blood agar (BA), one of MacConkey agar (MCA) and one of bile–esculin agar (BAE). One BA plate and the MCA and BAE plates were incubated at 37 °C ± 1 °C for 24 h in an aerobic environment, while one BA plate was incubated at 37 °C for 24 h in 5% CO_2_. Individual bacteria species were identified phenotypically and using routine biochemical tests, as specified in our internal laboratory procedures. In the case of three or more different, concomitant bacteria species without a prevalent one, the results were assigned as “polymicrobism”.

### 2.3. Statistical Analysis

Continuous variables were summarized as mean ± standard deviation (SD), and categorical variables were reported as frequencies or percentages. The pre- and post-laser treatment bacteria counts were transformed (by logarithm base 10), and these were compared using non-parametric techniques, specifically the Wilcoxon test, given the limited number of samples processed. A *p*-value < 0.05 was deemed to indicate statistical significance for the analysis, which was conducted using the online software Stata BE 17.3 (StataCorp LLC, College Station, TX, USA).

## 3. Results

Small-breed dogs were the most representative group studied: four miniature Poodles, four toy Poodles, a Chihuahua, a Shih Tzu, a Jack Russell Terrier, and two small to medium-sized mixed-breed dogs were enrolled. The study population consisted of 50% male dogs and 50% female dogs, with a mean age of 4.7 ± 3.4 years old. The type of diet consumed by the animals was recorded, with the following categories observed: dry food, wet food, home-cooked food, or a combination of these (Table 1).

A higher mean mesophilic bacteria count was found in gingival swabs prior to laser application than in the post-laser treatment swabs. The mean number of bacteria per swab pre-laser treatment was 2,007,500 ± 2,631,073 colony-forming units (CFUs), while after treatment, it was 716,515.6 ± 1,178,972. The results of the study demonstrated that laser therapy was effective in reducing bacterial loads in all the subjects except two, with a mean bacteria count decrease of 65.7 ± 10.5% after the MLS application.

**Figure 1:**
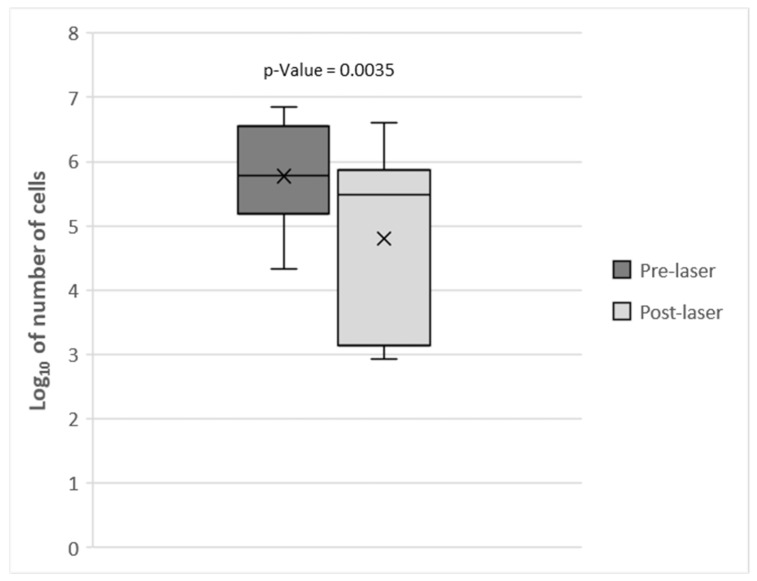
Mesophilic bacteria counts (log_10_CFU/swab) in swabs from the teeth of 16 dogs pre- and post-laser treatment. Means are indicated by × and standard deviations by bars.

On the logarithmic scale, the mean mesophilic bacteria count was reduced by 1 logarithm unit by the laser treatment: before the laser treatment, the mean bacteria count was 5.77 ± 0.21 log_10_CFU/swab (n = 0.44; (95% confidence interval (CI)), while after laser treatment, it was 4.80 ± 0.346 log_10_CFU/swab (n = 0.73; 95% CI). The statistical analysis revealed that this difference was highly significant (*p* = 0.035) (Figure 1).

A polymicrobial result was obtained in five cases from both gingival swabs. In three cases, a *Pseudomonas* species was isolated, while *Escherichia coli* was isolated in one case. In one case, *Escherichia coli* and Enterobacteriaceae species were isolated from the gingival swab before and after the laser treatment, respectively (Table 2).

## 4. Discussion

Laser therapy could be a viable tool in standard oral hygiene sessions for the prevention and treatment of periodontal disease, based on the effectiveness demonstrated in various studies [26,27,28,39,40]. The present study demonstrated that laser therapy was effective in 80% of the subjects, producing a mean bacteria count decrease of 65.7 ± 10.5% after MLS treatment. A significant 1-log reduction in gingival bacteria count was observed in the dog population following the laser treatment (compared with before treatment), indicating that laser therapy could be a valuable tool for bacterial decontamination, as previously reported [39,41]. In a study conducted by Fontana et al. (2004), it was observed that the application of laser radiation resulted in a significant reduction in bacteria in rats with periodontal disease [27]. The absence of bacterial reduction in two animals may be due to the innate resistance of certain bacterial strains or the presence of contaminated saliva in the gingival pouch prior to or during the post-treatment sampling. The duration of the bacteria reduction and the potential for further laser treatments to enhance this reduction remain subjects for further investigation. It can be hypothesized that the laser application induced an increase in the temperature at the site where the laser was utilized, leading to the bacteria reductions.

Microbiological analyses were performed on a subgroup of 10 dogs because qualitatively, no significant effects were observed. In almost all cases, the same bacteria species grew from gingival swabs collected before and after the laser treatment. In a single instance, *Escherichia coli* was solely identified from a swab collected prior to the laser treatment, whereas Enterobacteriaceae were cultured from the swab collected after the laser treatment. It can be postulated that the laser treatment resulted in a significant reduction in *Escherichia coli* presence, thereby facilitating the proliferation of other species. It is also possible that significant changes in the bacteria species present were not observed due to the limited number of dogs included in this investigation. For this study, the authors preferred to investigate the effects induced by MLS laser treatment on mesophilic bacteria rather than focusing on effects on any single bacteria species, considering that dog periodontal disease occurs worldwide in many dog breeds, and therefore the bacteria in dogs’ gingival sulcus could differ greatly. Subsequent work should be carried out by applying culture procedures that allow for the growth of bacteria with specific nutritive requirements, such as *Porphyromonas* spp. and *Peptostreptococcus canis* that are frequently involved in periodontal disease.

The healing effect produced by laser treatment was not evaluated in this study. However, it was reported that laser treatment contributes to the reduction in inflammation and erythema [40], which favors periodontal disease prevention. Khadra et al. (2004) reported an improvement in tissue healing in an experimental study [28]. Periodontal disease is a significant challenge in small animal practices and has a high prevalence of clinical manifestations [42,43]. Despite the investigation of several risk factors for periodontal disease, many aspects remain unknown. Further studies are required to elucidate the potential contributions of other factors to the development of periodontal disease in dogs and to identify strategies for its prevention and treatment, with the objective of reducing its prevalence. Dental home care is essential to prevent periodontal disease in dogs, but it should be accompanied by regular professional interventions. To the best of our knowledge, this is the first study that investigated MLS laser application in periodontal disease. MLS laser treatment does not supplant the necessity for daily home oral hygiene practices; however, it could offer an additional tool for the control of periodontal disease, given its ease of use, patient tolerability, painlessness, and lack of need for sedation. Further studies are required to characterize the effects of the MLS laser treatment and its usefulness for periodontal disease management in dogs.

## 5. Conclusions

This study demonstrated the efficacy of MLS laser treatment in reducing the bacterial load present within the gingival sulcus in dogs. The MLS laser treatment should be considered a supplementary measure for controlling dental plaque in dogs. It should not be viewed as a replacement for daily home oral hygiene practices or veterinary medical procedures for a dog’s oral health. Rather, it is an additional resource for the control of periodontal disease.

## Figures and Tables

**Table 1:** Anamnestic and clinical data of the dog population enrolled in the study.

Subject ID	Race	Sex	Age (Years Old)	Diet	Severity of Periodontitis
1	Toy Poodle	M	5.5	Dry Food + Wet Food	Mild
2	Miniature Poodle	F	11	Dry Food + Wet Food	Moderate
3	Miniature Poodle	F	5.5	Dry Food + Wet Food	Moderate–Severe
4	Miniature Poodle	M	8	Dry Food + Wet Food	Mild–Moderate
5	Toy Poodle	F	3.5	Dry Food + Wet Food + Home-cooked food	Moderate
6	Chihuahua	M	3.5	Dry Food + Wet Food	Moderate–Severe
7	Toy Poodle	F	2.5	Dry Food + Wet Food + Home-cooked food	Moderate
8	Miniature Poodle	M	7	Wet Food + Home-cooked food	Severe
9	Toy Poodle	M	4.7	Dry Food + Wet Food + Home-cooked food	Moderate–Severe
10	German Shepherd	M	2.5	Dry Food	Moderate
11	Border Collie	M	0.58	Dry Food	Absent
12	Czechoslovakian Wolfdog	F	4	Wet Food	Moderate
13	Jack Russell Terrier	F	1	Dry Food	Mild–Moderate
14	Mixed breed	F	11	Dry Food + Wet Food + Home-cooked food	Moderate
15	Mixed breed	F	0.5	Dry Food	Moderate
16	Shih Tzu	M	4.5	Home-cooked food	Moderate–Severe

**Table 2:** Results of microbiological cultures of the gingival swabs collected from a subgroup of ten dogs pre- and post-laser treatment.

Subject ID	Isolated Microorganisms Pre-Treatment	Isolated Microorganisms Post-Treatment
1	Polymicrobism	Polymicrobism
2	Polymicrobism	Polymicrobism
3	Polymicrobism	Polymicrobism
4	*Escherichia coli*	*Escherichia coli*
5	Polymicrobism	Polymicrobism
6	*Escherichia coli*	Enterobacteriaceae
7	*Pseudomonas* *aeruginosa*	*Pseudomonas* *aeruginosa*
8	*Pseudomonas* spp.	*Pseudomonas* spp.
9	*Pseudomonas* spp.	*Pseudomonas* spp.
10	Polymicrobism	Polymicrobism

## Data Availability

The raw data supporting the conclusions of this article will be made available by the authors on request.
